# Measuring interictal burden among people affected by migraine: a descriptive survey study

**DOI:** 10.1186/s10194-022-01467-z

**Published:** 2022-08-08

**Authors:** Lena T. Hubig, Timothy Smith, Emma Williams, Lauren Powell, Karissa Johnston, Linda Harris, Gilbert L’Italien, Vladimir Coric, Andrew J. Lloyd, Siu Hing Lo

**Affiliations:** 1 Acaster Lloyd Consulting Ltd, 8th Floor, Lacon House, 84 Theobalds Road, London, WC1X 8NL UK; 2 StudyMetrix LLC, 3862 Mexico Road, St. Peters, MO 63303 USA; 3 Broadstreet HEOR, 201 – 343 Railway Street, Vancouver, British Columbia V6A 1A4 Canada; 4 Biohaven Pharmaceuticals Inc, 215 Church Street, New Haven, CT 06510 USA

**Keywords:** Migraine, Patient-reported outcome, Questionnaire, Health-related quality of life, Disease burden

## Abstract

**Background:**

Previous research has extensively documented the impact of migraine episodes (‘ictal’) on patients’ health-related quality of life. Few studies have looked at the impact of migraine on migraine-free days (‘interictal’). This study was designed to describe interictal burden of migraine in a mixed group of people affected by migraine and to explore patient characteristics associated with interictal burden.

**Methods:**

People with migraine in the United States (US) and Germany were recruited for a cross-sectional online survey, including a subgroup treated with calcitonin gene-related peptide (CGRP) monoclonal antibody (mAb). The survey included the Migraine Interictal Burden Scale (MIBS-4), Headache Impact Test (HIT-6), and items measuring patient demographics, clinical and treatment background. Data were analyzed using descriptive statistics and linear regression.

**Results:**

Five hundred six people with migraine completed the survey (US: *n* = 257; Germany: *n* = 249), of whom 195 had taken a CGRP mAb for three or more months. Participants had a mean of 8.5 (SD = 6.4) Monthly Migraine Days (MMD) and 10.4 (SD = 7.1) Monthly Headache Days (MHD). The mean MIBS-4 score was 6.3 (SD = 3.4), with 67% reporting severe interictal burden (MIBS-4: ≥5). The mean HIT-6 score was 65.3 (SD = 6.0), with 86% reporting severe migraine impact (HIT-6: ≥60). MIBS-4 was correlated with the HIT-6 (*r* = 0.37), MMD and MHD (both *r* = 0.27). The HIT-6, MMD, MHD, CGRP mAb treatment, and depression all had an independent positive association with the MIBS-4.

**Conclusion:**

Two-thirds of the study sample reported substantial interictal burden. Whilst interictal burden was associated with migraine frequency and impact of migraine attacks, study results also show it represented a distinct aspect of the overall disease burden. Study findings further indicate unique associations between interictal burden and depression. A unique positive association between interictal burden and CGRP mAb treatment suggests a remaining unmet need among people affected by migraine treated with CGRP mAb**.**

## Background

Migraine is a debilitating neurovascular disorder that affects over 1 billion people worldwide [1]. The condition is characterized by attacks of severe head pain which can be accompanied by a range of other symptoms including nausea and sensitivity to light and sound [2]. Other symptoms such as tiredness and irritability can precede the headache by several days [3], and most attacks are followed by periods of feeling unwell, usually with symptoms such as tiredness, brain fog and stiff neck [4, 5]. Migraine phases are not mutually exclusive, and symptoms associated with one phase may overlap with or endure into different phases [6].

Many migraine treatments are available, both for acute and preventive use, yet patient satisfaction with treatment is often poor [7, 8]. Conventional preventive treatments, such as antiepileptics, antidepressants or betablockers, are associated with side effects, limited effectiveness, and low adherence [8]. Novel calcitonin gene-related peptide monoclonal antibody (CGRP mAb) treatments are approved for the preventive treatment of migraine. These treatments have been shown in clinical trials to be safe and effective in reducing migraine frequency [9–12], but patients still experience breakthrough attacks and use acute abortive therapy [13, 14].

Despite the availability of a large number of available treatments, the disease burden of people affected by migraine remains high, and a large body of research has demonstrated the substantial functional impairments and impact on patients’ health-related quality of life (HRQL) caused by migraine [15–18]. The impact of migraine symptoms on patients physical and cognitive ability as well as psychosocial and emotional well-being have been extensively documented [15, 18–22].

A growing body of evidence shows that migraine episodes cause impairments between attacks (interictal burden). In this study, we follow the definition of interictal burden (in the context of migraine) defined by Lampl et al. as the ‘loss of health or wellbeing attributable to a headache disorder reportedly experienced while headache-free’ that can affect all areas of life on any day [23]. In a recent qualitative study, patients described behavioral changes and adaptations, strained relationships, social isolation, work and career impacts, and emotional impacts, including feeling helpless, unreliable, and anxious in anticipation of the next attack when migraine free [24]. Previous studies have also shown that interictal burden is associated with psychological disorders such as anxiety and depression, and reduced workplace productivity [25, 26]. Migraine frequency and ictal burden have been associated with interictal burden [25, 27], and one recent clinical trial of galcanezumab demonstrated that treatment lowered interictal burden compared with placebo [28]. Nevertheless, studies have also demonstrated that interictal burden is distinct from other constructs relating to the impact of migraine on HRQL [25, 28], and even patients with few migraine days can experience interictal burden [27].

The Headache Impact Test (HIT-6) has been widely used in clinical research and management of migraine to quantify migraine burden, assessing pain severity and the impact of migraine attacks on daily activities, fatigue, emotional wellbeing, and cognition [29–32]. A less frequently used validated instrument is the Migraine Interictal Burden Scale (MIBS-4) [33], which is to our knowledge the only instrument specifically developed to measure the interictal burden of migraine. The instrument measures impairment in work or school, impairment in family and social life, difficulty in making plans or commitments, and emotional/affective and cognitive distress on days without migraine [25, 33].

This study was designed to describe the interictal burden as measured by the MIBS-4 in a mixed sample of people with migraine with and without a chronic migraine diagnosis and/or experience with CGRP mAb treatments, and to explore the relationship between migraine interictal burden and patient demographic, clinical and treatment background.

## Methods

### Study design

This study on migraine interictal burden formed part of a larger online survey study developed to examine patients’ burden of disease, treatment experience, and treatment preferences for preventive migraine treatments.

People with a self-reported medical diagnosis of migraine were recruited between September and November 2021 to participate in a cross-sectional online survey. Potential participants were identified by a specialist recruitment agency through commercial databases (patient panels). Participants were eligible to take part if they had a (self-reported) diagnosis of migraine from a medical doctor, resided in the United States (US) or Germany, and were at least 18 years old. Potential participants were screened, and, if eligible, provided with further details regarding the study and their rights as study participants. If they consented to participate in the study, they were directed to the main survey. The target sample size was set to 500 (250 per country) to support the study objective of quantifying patient treatment preferences [34]. A minimum quota was set for patients who had taken a CGRP mAb for at least 3 months, the minimum required treatment duration to assess clinical benefit as reported in the literature(100 in the US; 50 in Germany) [35].

The study was reviewed and approved by WCG Institutional Review Board (Study Number: 1305360; IRB Tracking Number: 20211304) before recruitment.

### Survey

Survey development was guided by a targeted literature review and qualitative interviews with people affected by migraine in the UK, US, and Canada [24]. The survey draft was finalized following input from cognitive debrief interviews with people with migraine (*n* = 5) and a review by a clinical migraine expert (TS).

The survey included standard validated measures of interictal burden (MIBS-4) and migraine impact (HIT-6), as well as bespoke survey items measuring patient characteristics. The MIBS-4 is designed to measure the interictal burden of migraine, i.e., the impact of migraine on patient life when they are not experiencing a migraine [26]. The instrument has a recall period of 4 weeks and contains four items, which are scored on a six-point Likert scale. The total score ranges from 0 to 12, where higher total scores indicate more severe interictal burden. The MIBS-4 total score indicates the following: no interictal burden (0), mild level (1, 2), substantial level (3, 4) and severe level (≥5) of interictal burden. The HIT-6 was developed and validated to capture the impact of migraine on patient’s life and its associated disease burden [19, 29]. Its six items are scored on a five-point Likert scale, with higher scores indicating more severe burden. The total score ranges from 36 to 78 and can be classified into little or no impact (≤49), some impact (50–55), substantial impact (56–59) and severe impact (≥60). Bespoke survey items measured sociodemographic and clinical characteristics of patients and their experience with acute and preventive treatments. The survey was developed in English for US participants and translated to German and adapted to account for differences between the two countries. For the HIT-6, the independently validated German language version was used [36]; the German translation of the MIBS-4 and bespoke survey items were reviewed by a native German speaker (LTH) fluent in English.

### Analysis

Sample characteristics were summarized descriptively for the overall sample and stratified by country (categorical variables: count and percentage; continuous variables: mean, standard deviation [SD]). Results of the MIBS-4 and HIT-6 were presented overall and stratified by CGRP mAb experience (CGRP mAb treatment for at least 3 months) and chronic migraine diagnosis (ever received a chronic migraine diagnosis by a doctor). Correlations between monthly migraine days (MMD), monthly headache days (MHD) and HIT-6 scores and MIBS-4 scores were described with Pearson’s correlation coefficients. As only completed surveys were recorded, there were no missing values.

To explore factors uniquely associated with the MIBS-4, multivariable linear regression models with the MIBS-4 score as dependent variable were estimated. The initial set of independent variables were selected based on background knowledge of the following previously described factors associated with interictal burden: migraine frequency [27, 28], the impact of migraine attacks [25, 28], employment [24, 25], anxiety/depression [23–25], and treatment effect [24, 28]. The study design and sample size were not informed by a priori hypotheses on differences in interictal burden between patients with different demographic or clinical profiles and effect sizes of any such hypothesized differences; thus, statistical significance ought to be understood as exploratory [37].

The final set of independent variables included MMD (continuous), MHD (continuous), HIT-6 score (continuous), employment status (employed full−/part-time/retired/other), other health conditions (depression: yes/no; anxiety: yes/no), patient satisfaction with their overall treatment regimen (yes/no), and CGRP mAb treatment experience (in the last 3 months/in the past/never). The final model also included sex (female/male), age (continuous) and country (US/Germany) to adjust for any demographic differences. Variables considered but excluded from the final model were chronic migraine diagnosis, due to overlap with migraine frequency and the migraine frequency variables corresponding more closely to the MIBS-4 recall period, and preventive (non-CGRP mAb) treatment experience, due to heterogeneity of patients’ medication experiences and overlap with CGRP mAb treatment.

The model was assessed for multicollinearity using the Variance Inflation Factor (VIF), and the stability of the estimators and variance in different sets of independent variables were tested; no adjustments for multicollinearity were necessary. Including an interaction term for MMD and MHD was also assessed but did not improve model fit (*p* = 0.70).

All analyses were conducted in R 4.1.2 [38].

## Results

### Sample characteristics

Overall, 10,075 individuals clicked on the survey link, of which 8250 (82%) were ineligible and 680 (7%) consented to participate. Five hundred six patients from the US (*n* = 257, 51%) and Germany (*n* = 249, 49%) completed the survey (Table 1). Participants had a mean (SD) age of 44.6 (13.7) years and a majority were (63%) female. A large proportion (70%) reported chronic health conditions in addition to migraine, most frequently anxiety (31%) and depression (29%).

**Table 1:** Patient demographic and clinical characteristics

Characteristic	Overall, ***N = 506***	US, ***n = 257***	Germany, ***n = 249***
**Age (years)**, mean (SD)	**45.0 (13.8)**	44.8 (14.7)	45.2 (12.9)
**Sex**			
Male	**184 (36%)**	110 (43%)	74 (30%)
Female	**322 (64%)**	147 (57%)	175 (70%)
**Employment status**			
Employed part−/full-time	**341 (67%)**	173 (67%)	168 (67%)
Retired	**96 (19%)**	48 (19%)	48 (19%)
Other	**69 (14%)**	36 (14%)	33 (13%)
**Disease duration (years)**^**a**^, mean (SD)	**18.2 (14.9)**	16.5 (15.0)	20.0 (14.6)
**Chronic migraine (ever diagnosed)**	**239 (47%)**	148 (58%)	91 (37%)
**Monthly migraine days**, mean (SD)	**8.7 (6.4)**	10.6 (6.8)	6.7 (5.2)
**Monthly headache days**, mean (SD)	**10.6 (7.1)**	12.2 (7.7)	8.8 (6.0)
**Preventive treatment (non-CGRP mAb)**			
In the last 3 months	**217 (43%)**	125 (49%)	92 (37%)
In the past	**141 (28%)**	84 (33%)	57 (23%)
Never	**148 (29%)**	48 (19%)	100 (40%)
**CGRP mAb treatment**			
In the last 3 months	**83 (16%)**	52 (20%)	31 (12%)
In the past	**112 (22%)**	78 (30%)	34 (14%)
Never	**311 (61%)**	127 (49%)	184 (74%)
**Satisfied with treatment regimen**^**b**^			
No	**297 (59%)**	151 (59%)	146 (59%)
Yes	**209 (41%)**	106 (41%)	103 (41%)
**Other chronic health condition – any** (yes)	**354 (70%)**	197 (77%)	157 (63%)
Physical health condition	**232 (46%)**	137 (53%)	95 (38%)
Mental health condition	**227 (45%)**	130 (51%)	97 (39%)
*Anxiety*	**159 (31%)**	109 (42%)	50 (20%)
*Depression*	**148 (29%)**	77 (30%)	71 (29%)
*Other*	**123 (24%)**	64 (25%)	59 (24%)

*CGPR* Calcitonin gene-related peptide, *mAb* Monoclonal antibody, *SD* Standard deviation

^a^Disease duration was calculated as years since first migraine symptoms, if known, otherwise years since migraine diagnosis (*n* = 19). Those who did not report years since first symptoms nor years since diagnosis were excluded (*n* = 20)

^b^Satisfied with all treatments (acute/preventive/CGRP mAb) taken

Patients had had migraine symptoms for a mean (SD) of 18.2 (14.9) years, and around half (47%) had been diagnosed with chronic migraine in their lifetime. Patients reported a mean of 8.7 (SD = 6.4) MMDs and 10.6 (SD = 7.1) MHDs in the previous 3 months.

The majority of participants took preventive treatments: 43% had taken a conventional (non-CGRP mAb) treatment and 16% had taken a CGRP mAb treatment in the last 3 months. The proportion of participants in Germany who have never taken a preventive treatment was higher than in the US (40% versus 19%).

Under half (41%) of the participants were satisfied with their treatment regimen, defined as satisfaction with acute treatments, preventive treatments and/or CGRP mAb treatments, as applicable.

### Interictal migraine burden

Table 2 shows the interictal burden and migraine impact as measured by the MIBS-4 and HIT-6 instruments respectively. Overall, participants had a mean score of 6.3 (SD = 3.4) on the MIBS-4. Most participants fell into the severe (MIBS-4 score: ≥5) interictal burden category (67%), and only 4% had no interictal burden (MIBS-4 score: 0). Patients with CGRP mAb experience and patients with chronic migraine were more likely to fall into the severe interictal burden category than non-mAbs patients (84% vs. 57%) and patients with non-chronic migraine (78% vs. 58%) respectively. On average, participants scored 65.3 (SD = 6.0) on the HIT-6 and the majority of participants fell into the severe (HIT-6 score: ≥60) impact category (86%).

**Table 2:** Migraine Interictal Burden Scale (MIBS-4) and Headache Impact Test (HIT-6) overall and stratified by CGRP mAb treatment experience (3+ months) and chronic migraine (CM) diagnosis

Characteristic	Overall, ***N = 506***	No CGRP mAb, ***n = 311***	CGRP mAb, ***n = 195***	No CM, ***n = 267***	CM, ***n = 239***
**Interictal burden**					
**MIBS-4 Score (0–12)**					
Mean (SD)	**6.3 (3.4)**	5.3 (3.3)	7.9 (3.1)	5.5 (3.4)	7.2 (3.3)
Level of interictal burden					
*None (0)*	**22 (4%)**	20 (6%)	2 (1%)	15 (6%)	7 (3%)
*Mild* *(1, 2)*	**72 (14%)**	60 (19%)	12 (6%)	51 (19%)	21 (9%)
*Moderate (3, 4)*	**72 (14%)**	54 (17%)	18 (9%)	47 (18%)	25 (10%)
*Severe (≥5)*	**340 (67%)**	177 (57%)	163 (84%)	154 (58%)	186 (78%)
**Headache/ migraine impact**					
**HIT-6 Scale (36–78)**					
Mean (SD)	**65.3 (6.0)**	65.1 (6.2)	65.7 (5.5)	64.3 (6.3)	66.5 (5.4)
Level of impact					
*Little or no (≤49)*	**5 (1%)**	4 (1%)	1 (1%)	4 (2%)	1 (0%)
*Some (50–55)*	**28 (6%)**	20 (6%)	8 (4%)	19 (7%)	9 (4%)
*Substantial (56–59)*	**39 (8%)**	26 (8%)	13 (7%)	29 (11%)	10 (4%)
*Severe (≥60)*	**434 (86%)**	261 (84%)	173 (89%)	215 (81%)	219 (92%)

*CGPR* Calcitonin gene-related peptide, *CM* Chronic Migraine, *HIT-6* Headache Impact Test, *mAb* Monoclonal antibody, *MIBS-4* Migraine Interictal Burden Scale, *SD* Standard Deviation

**Fig. 1:**
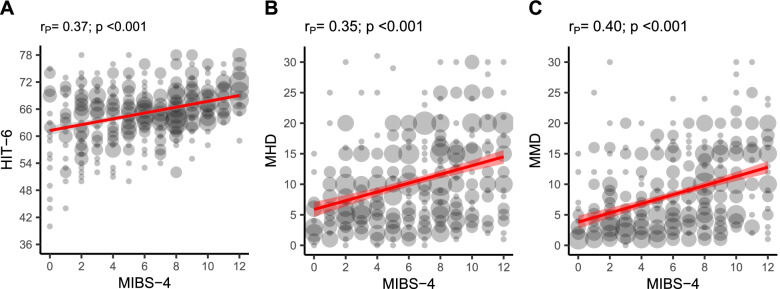
Relationship between MIBS-4 and **A** HIT-6, **B** MHD, and **C** MMD. The estimated linear association and 95% CI between the two variables are shown in red. Bubble size is proportionate to the number of observations

There was a moderate positive correlation between the MIBS-4 score and HIT-6 scale (*r* = 0.37, *p* < 0.001), suggesting that patients with a higher (ictal) migraine impact also had a higher interictal burden (Fig. 1). MMDs and MHDs showed a small positive correlation with the HIT-6 score (both: *r* = 0.27, *p* < 0.001) and a moderate positive correlation with MIBS-4 (*r* = 0.40, *p* < 0.001; *r* = 0.35, *p* < 0.001).

**Fig. 2:**
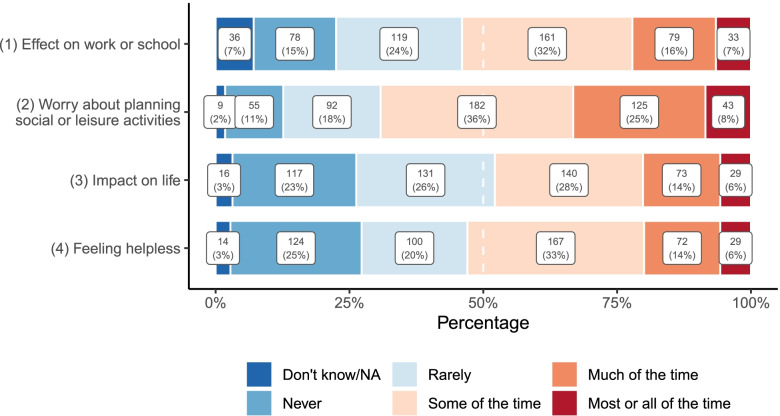
Number (Percentage) of participants reporting MIBS-4 item-level impairment

Figure 2 shows participants’ item-level responses on the MIBS-4. Most (69%) participants stated they worry about planning social or leisure activities because they may have a headache (Item 2) at least some of the time, and one-third (33%) stated they worried about this much, most or all of the time. In addition, over half (53%) of participants agreed that their headaches affect their work or school (Item 1) and feeling helpless (Item 4) when they do not have a headache at least some of the time. Slightly under half (48%) agreed that headaches impact their life when they do not have a headache (Item 3) at least some of the time.

Results from the final multivariable regression model are presented in Table 3. The regression model confirms the correlation results as it suggests an independent positive association between HIT-6 and migraine/headache frequency: The MIBS-4 score increased by 0.09 (SE = 0.03) for each additional MMD, by 0.07 (SE = 0.02) for each additional MHD, and by 0.14 (SE = 0.02) for each additional point on the HIT-6 (range: 36–78).

**Table 3:** Multivariable linear regression model of MIBS-4 scores (interictal burden). A positive estimate suggests the characteristic is associated with worse interictal burden; a negative estimate suggests it is associated with lower interictal burden

Characteristic	Estimate	SE	95% CI^**a**^	***p***-value^**a**^
**HIT-6 score**	0.14	0.02	0.10, 0.19	< 0.001
**Monthly migraine day**	0.09	0.03	0.04, 0.15	< 0.001
**Monthly headache day**	0.07	0.02	0.02, 0.12	0.003
**Depression**				
No	0.00	–	–	–
Yes	0.74	0.34	0.08, 1.40	0.03
**Anxiety**				
No	0.00	–	–	–
Yes	0.00	0.34	−0.67, 0.66	> 0.9
**Employment status**				
Part−/Full-time	0.00	–	–	–
Other^b^	− 0.41	0.35	−1.09, 0.27	0.24
Retired	−0.72	0.44	−1.59, 0.15	0.11
**Satisfied with treatment regimen**^**c**^				
No	0.00	–	–	–
Yes	−0.30	0.26	−0.81, 0.20	0.24
**CGRP mAb treatment (3+ months)**				
In the last 3 months	0.00	–	–	–
In the past	0.69	0.41	−0.11, 1.50	0.09
Never	−1.42	0.37	−2.14, −0.69	< 0.001
**Sex**				
Male	0.00	–	–	–
Female	−0.37	0.27	−0.91, 0.17	0.18
**Age (years)**	−0.02	0.01	−0.04, 0.01	0.15
**Country**				
US	0.00	–	–	–
Germany	0.48	0.28	−0.07, 1.03	0.09

R^2^ = 0.358; Adjusted R^2^ = 0.341; Statistic = 21.1; *p*-value = < 0.001; AIC = 2490; BIC = 2554; *N* = 506

*CGRP* Calcitonin-gene related peptide, *CI* Confidence Interval, *HIT-6* Headache Impact Test, *mAb* monoclonal antibody, *SE* Standard Error

^a^95% CIs and *p*-values are exploratory

^b^Unemployed, unable to work due to health issues, student, homemaker, other

^c^Yes: Satisfied or very satisfied with all treatments taken (acute/preventive/CGRP mAb treatment)

The model also explored the independent effect of migraine treatments on interictal burden. Compared to patients who had taken CGRP mAb treatment in the last 3 months, patients who had never taken a CGRP mAb treatment had less interictal burden (− 1.42, SE = 0.37), even after adjusting for migraine frequency (MMD, MHD) and impact of migraine attacks (HIT-6). In contrast, patients who had taken CGRP mAb in the past had worse interictal burden (0.69, SE = 0.41) than patients who had taken CGRP mAb treatment in the last 3 months. In addition, being satisfied with their overall treatment regimen may be uniquely associated with lower interictal burden (− 0.30, SE = 0.26).

The model estimate suggests that depression is uniquely associated with worse interictal burden and increase the MIBS-4 score by 0.74 (SE = 0.34) points but found no change in MIBS-4 score for anxiety (0.0, SE = 0.34). Further, the results suggest a negative relationship between employment and interictal burden: Patients who were retired were predicted to have an average of − 0.72 (SE = 0.44) lower MIBS-4 score than employed patients, independent of age.

## Discussion

This study assessed interictal burden and impact of migraine attacks in a sample of people with variable migraine disease burden and a subset of patients with CGRP mAb treatment experience. The study highlighted the severe disease burden people affected by migraine experience even when not having an acute migraine episode.

Two-thirds of the overall study sample had severe interictal burden, as measured by the MIBS-4. Furthermore, over half reported severe interictal burden irrespective of whether they had or did not have a chronic migraine diagnosis or whether they had or had not been treated with CGRP mAb. Following the recommendations of the MIBS-4 instrument developers [25], 81% of the total study sample should be considered for preventive treatment. The prevalence of moderate to severe interictal burden in the current study was higher than in a previous international study exploring migraine burden in patients (81% vs. 28%) [39]. Differences in prevalence of interictal burden may have been a result of the current study including patients with a medical diagnosis of migraine only, while the previous study included both patients with and without a medical diagnosis.

Our findings show that impact of migraine attacks, as measured by the HIT-6, migraine frequency and headache frequency were each uniquely associated with interictal burden. Previous studies have also shown a positive, albeit weaker, association between interictal burden and migraine/headache frequency [27, 28]. This study showed a positive association between ictal disability (HIT-6) and interictal burden (MIBS-4) but did not explore specific biological or clinical drivers of interictal burden. Future studies could investigate the impact of ictal symptoms, for example nausea or photo−/phonophobia, and pre- and postictal symptoms of the pro- and postdrome phase on interictal burden, on interictal burden. This may help better understand patients’ unmet need for acute and/or preventive migraine treatment and optimize treatment plans.

Our study also explored the unique association between interictal burden and more distal demographic, clinical and treatment factors. First, depression was associated with worse interictal burden. Qualitative evidence suggests that feelings of depression due to secondary impacts of migraine (e.g., being unreliable, unable to plan, having to abandon social activities) are how the migraine interictal burden can manifest [24]. Consistent with this hypothesis, the item-level results of the MIBS-4 also showed that worry about social and leisure activity was where most patients experienced impairment.

Second, patients who were treated with a CGRP mAb in the last 3 months had lower interictal burden than patients treated with CGRP mAbs in the past. However, patients who were treated with a CGRP mAb in the last 3 months had more severe interictal burden than patients who had never received a CGRP mAb, even after adjusting for migraine frequency and impact of migraine attacks. Patients who are treated with CGRP mAb are commonly thought to be more severely impacted than other patients, as they need to qualify for CGRP mAb treatment, for example by having refractory disease [40, 41]. To our knowledge, interictal burden is not regularly considered when treatment benefits are assessed, and the relationship between treatment and interictal burden is not well understood. One recent galcanezumab clinical trial included the MIBS-4 as a secondary outcome measure and was able to show a treatment benefit [28]. Our study results add to this by showing that patients who were taking CGRP mAb in the last 3 months still have higher interictal burden than patients who have never taken CGRP mAb. Further research investigating the effect of CGRP mAb and other treatments on interictal burden is necessary to fully understand how treatments may relieve interictal burden. Considering the high levels of interictal burden found in this study, these findings indicate that many patients on CGRP mAb have a remaining unmet need.

The study results showed a tentative unique association between being employed and more severe interictal burden, with retired patients being the least affected. This might be due to the unpredictable nature of migraine and being employed may cause additional worry associated with migraine, in line with qualitative findings [24] and item 1 of the MIBS-4, which specifically explores the interictal effect of migraine on work. The impact of migraine attacks on work and productivity is well established [42, 43]. Our study findings suggest that the impact of migraine may go beyond the immediate productivity loss due to migraine days, as interictal burden may also be associated with reduced productivity and absenteeism [23, 44].

Our results also indicate a tentative unique association between satisfaction with the overall treatment regimen and less interictal burden. Having no control over migraine has been described as an important aspect of interictal burden [23], and having access to reliable treatment could give patients more control over their migraine, thus reducing their interictal burden.

### Strengths and limitations

The study recruited a large sample of people with a self-reported diagnosis of migraine in the US and Germany. The minimum quota set for patients treated with CGRP mAb resulted in a study sample with relatively severe disease burden and the sample may underrepresent those with milder migraine. Due to the cross-sectional and exploratory study design, no a priori hypotheses on relationships between interictal burden and patient characteristics were tested, and it was not possible to determine causality. Given the study design and recruitment from patient panels, the results of the study may not be representative of the population of people affected by migraine.

While the HIT-6 is validated in German and US people with migraine, the German translation of the MIBS-4 was not validated and the psychometric properties of the MIBS-4 across countries have not been studied. The translated study materials were reviewed and checked for accuracy by a native German speaker fluent in English. Further, the MIBS-4 has a recall period of 4 weeks, while other explanatory variables specified different time periods, leading to unprecise estimates.

This study relies on self-reports of participants which can bias the findings. Participants may have had difficulty recalling their number of migraine or headache days and provided inaccurate estimates. Due to the study design, it was not possible to verify participant responses; in particular use of CGRP mAb treatments and diagnosis of reported conditions (i.e. migraine, depression and anxiety) were not verified by a clinician.

For these reasons, the results of the multivariable regression analysis are presented to generate hypotheses and support further research in examining factors contributing to or alleviating interictal burden [37].

## Conclusion

This study found a substantial interictal burden in over half of the study sample, for patients with and without chronic migraine and irrespective of patient CGRP mAb treatment experience. The study findings also indicate that interictal burden is associated with migraine frequency and the impact of migraine attacks, whilst representing a distinct aspect of the overall burden of migraine. The results further highlight the unique association between interictal migraine burden and depression, and a remaining unmet need among patients treated with CGRP mAb treatments, generating hypotheses that could be examined in future studies. To conclude, the study findings on interictal burden help to describe the overall disease burden of migraine, a chronic disorder characterized by recurring and often unpredictable attacks that can impair patients’ lives at any time.

## Data Availability

The datasets used and/or analysed during the current study are available from the corresponding author on reasonable request.

## Abbreviations

AIC: Akaike Information Criterion
BIC: Bayesian Information Criterion
CGRP: Calcitonin Gene-related Peptide
CI: Confidence Interval
CM: Chronic Migraine
HIT-6: Headache Impact Test
HRQL: Health-related Quality of Life
mAb: monoclonal Antibody
MHD: Monthly Headache Day
MIBS-4: Migraine Interictal Burden Scale
MMD: Monthly Migraine Day
SD: Standard Deviation
SE: Standard Error
US: United States
